# VEGFR2 blockade inhibits glioblastoma cell proliferation by enhancing mitochondrial biogenesis

**DOI:** 10.1186/s12967-024-05155-1

**Published:** 2024-05-03

**Authors:** Min Guo, Junhao Zhang, Jiang Han, Yingyue Hu, Hao Ni, Juan Yuan, Yang Sun, Meijuan Liu, Lifen Gao, Wangjun Liao, Chunhong Ma, Yaou Liu, Shuijie Li, Nailin Li

**Affiliations:** 1 Department of Radiology, Beijing Tiantan Hospital, Capital Medical University, Beijing, China; 2 Department of Medicine-Solna, Division of Cardiovascular Medicine, Karolinska University Hospital, Solna, 171 76 Stockholm, Sweden; 3 Department of Oncology, Nanfang Hospital, Southern Medical University, Guangzhou, China; 4 Department of Biopharmaceutical Sciences and National Key Laboratory of Frigid Zone Cardiovascular Diseases (NKLFZCD), College of Pharmacy, Harbin Medical University, Harbin, China; 5 Department of Gynaecology and Obstetrics, Nanfang Hospital, Southern Medical University, Guangzhou, 510515 China; 6 Department of Cell and Molecular Biology, Karolinska Institutet, Stockholm, Sweden; 7 Department of Immunology and Shandong University-Karolinska Institutet Collaborative Laboratory, Shandong University Cheeloo Medical College, School of Basic Medicine, Jinan, China

**Keywords:** Glioblastoma, Vascular endothelial growth factor receptor 2, Mitochondria, Mitochondrial transcription factor A, Peroxisome proliferator-activated receptor gamma coactivator 1-α/PGC1α, Reactive oxygen species

## Abstract

**Background:**

Glioblastoma is an aggressive brain tumor linked to significant angiogenesis and poor prognosis. Anti-angiogenic therapies with vascular endothelial growth factor receptor 2 (VEGFR2) inhibition have been investigated as an alternative glioblastoma treatment. However, little is known about the effect of VEGFR2 blockade on glioblastoma cells per se.

**Methods:**

*VEGFR2* expression data in glioma patients were retrieved from the public database TCGA. VEGFR2 intervention was implemented by using its selective inhibitor Ki8751 or shRNA. Mitochondrial biogenesis of glioblastoma cells was assessed by immunofluorescence imaging, mass spectrometry, and western blot analysis.

**Results:**

*VEGFR2* expression was higher in glioma patients with higher malignancy (grade III and IV). VEGFR2 inhibition hampered glioblastoma cell proliferation and induced cell apoptosis. Mass spectrometry and immunofluorescence imaging showed that the anti-glioblastoma effects of VEGFR2 blockade involved mitochondrial biogenesis, as evidenced by the increases of mitochondrial protein expression, mitochondria mass, mitochondrial oxidative phosphorylation (OXPHOS), and reactive oxygen species (ROS) production, all of which play important roles in tumor cell apoptosis, growth inhibition, cell cycle arrest and cell senescence. Furthermore, VEGFR2 inhibition exaggerated mitochondrial biogenesis by decreased phosphorylation of AKT and peroxisome proliferator-activated receptor gamma coactivator 1-alpha (PGC1α), which mobilized PGC1α into the nucleus, increased mitochondrial transcription factor A (TFAM) expression, and subsequently enhanced mitochondrial biogenesis.

**Conclusions:**

VEGFR2 blockade inhibits glioblastoma progression via AKT-PGC1α-TFAM-mitochondria biogenesis signaling cascade, suggesting that VEGFR2 intervention might bring additive therapeutic values to anti-glioblastoma therapy.

**Supplementary Information:**

The online version contains supplementary material available at 10.1186/s12967-024-05155-1.

## Introduction

Glioblastoma (GBM) is the most common malignant brain tumor [1]. Current treatment includes debulking surgery followed by chemotherapy and radiotherapy; the five-year survival rate of GBM patients is, however, very dismal, with median survival of 14.6 months [2]. Robust neoangiogenesis, intratumoral heterogeneity and tumor mircroenviroment are hallmarks for tumor malignancies and contribute to their phenotypic plasticity and therapeutic resistance [3–6].

VEGFR2 (also known as kinase domain region (KDR), or fetal liver kinase-1 (FLK1)) is a tyrosine kinase receptor essential for VEGF-mediated physiological responses in endothelial cells. It has been shown that VEGF and its receptors VEGFR1 and VEGFR2 are important in glioma angiogenesis and proliferation of glioma cells [7]. Blockade of VEGF pathway could alleviate tumor vessels, decrease brain oedema, and improve the outcome of chemo- and radio- therapies. However, bevacizumab (an anti-VEGFA antibody) had limited improvement in overall survival in glioblastoma patients and was associated with higher adverse events, although it increased progression-free survivals [8–10], hence the underlying mechanisms mediated by VEGFR need to be further explored.

In addition to endothelial cells, growing evidence suggests that VEGF and VEGFR play important roles on tumor cell biology through the actions of autocrine, paracrine, and even “intracrine”, and that tumor-secreted VEGF provides pro-survival signaling through tumor cell-expressed VEGFR. These findings have been reported in various cancers, such as breast cancer cells [11], skin cancer cells [12], colorectal cancer cells [13] and glioblastoma stem-like cells [14]. VEGFR2 is expressed in GBM cells, with particular high-expression in EGFRvIII-positive glioblastoma cells. VEGFR2 ligation inhibits cellular senescence and promotes tumor progression [15, 16]. VEGFR2 blockade suppressed cell proliferation and increased cellular senescence [17, 18].

In the light of our recent study showing that VEGFR2 blockade hampered breast cancer cell proliferation via enhancing mitochondria biogenesis [19], herein, we aimed to investigate whether the expression of VEGFR2 correlates with the grading of gliomas and if VEGFR2 blockade-regulated mitochondria biogenesis operates as a general anti-cancer mechanism using the glioblastoma cells U38 and U87. We found that VEGFR2 expression was higher in grade III and IV glioma patients than that in grade II patients, and that VEGFR2 blockade inhibits glioblastoma cell growth via AKT-PGC1α-TFAM-mitochondria biogenesis signaling cascade. Our findings highlight the role of VEGFR2 in glioblastoma cells, which is executed independently from angiogenesis.

## Materials and methods

### Glioma patient data

Data of 636 glioma patients were retrieved from the public database The Cancer Genome Altas (TCGA), including 223 Grade II, 245 Grade III and 168 grade IV. Gene expression of *VEGFR2* (*KDR*) was compared among different grades of tumors. The K-M survival curve was made by using Graphpad software.

### Cancer cell cultures

Human glioblastoma multiforme cancer cell line U87 was purchased from the American Type Culture Collection (ATCC; Wesel, Germany), and U38 cells were characterized in Professor Monica Nister laboratory at Karolinska Institutet [20, 21]. The cell lines have been authenticated using Short Tandem Repeat (STR) profiling within the last three years. U87 cells were cultured using Dulbecco’s Modified Eagle Medium (DMEM; Thermo Fisher Scientific, Waltham, MA, USA) containing 10% fetal bovine serum (FBS, heat inactivated) and 1% penicillin–streptomycin at 37 °C with 5% CO_2_. U38 cells were cultured using Minimum Essential Medium (MEM; Gibco) containing 10% FBS and 1% penicillin–streptomycin at 37 °C with 5% CO_2_. Briefly, the U87 and U38 cells were seeded in 6-well plates at the density of 1.5 × 10^5^ cells per well. After overnight attachment, the cell media were replaced by DMEM or MEM containing vehicle (0.01% dimethyl sulfoxide) or Ki8751 (Tocris Bioscience, Bristol, UK). The cells were then cultured for 24, 48 or 72 h before further experiments. All experiments were performed with mycoplasma-free cells.

### Ki8751 drug sensitivity test

Glioblastoma cell sensitivity to Ki8751 was tested by the addition of Ki8751 at different concentrations and assessed by methylthiazolyldiphenyl-tetrazolium bromide (MTT) assay. Thus, U87 and U38 cells were seeded into 96-well plates at 1 × 10^4^ cells per well. After one-day culture, Ki8751 was added at a range of concentrations (0, 0.04, 0.08, 0.15, 0.3, 0.6, 1.25, 2.5, 5, 10 µM) and in triplicates on each condition for 48 h. Afterwards, the cell numbers were measured by MTT assay.

### Apoptosis and cell proliferation assays

The harvested cells were using trypsin-ethylene diamine tetraacetic acid (EDTA) solution and washed twice with ice-cold Dulbecco's phosphate buffered saline (DPBS). The cells were then stained with FITC-conjugated Annexin V and propidium iodide (PI) using a commercial cell apoptosis kit (V13241; Invitrogen) in the dark at room temperature and according to the manufacturer’s protocol. Cell proliferation was assessed using the cell counting kit (CCK)-8 assay (Dojindo Molecular Technologies; Rockville, MD, USA).

### Mass spectrometry analysis

The U87 cells were seeded in a 6-well-plate and treated with dimethyl sulfoxide (DMSO) or 2.5 µM Ki8751 for 24 h. After treatment, cells were lysed by RIPA buffer (Invitrogen) supplied with phosphatase and protease inhibitors (Rhoche) on ice for 20 min. Each sample (50 µg proteins) was reduced with 10 mM DTT (Sigma; #D0632) at 55 °C for 45 min and then alkylated with 25 mM IAA (Sigma; # I6125) at room temperature for 30 min in the dark. Acetone was used to precipitate the proteins overnight, and the precipitation was dissolved in 15 μL of 8 M urea (Sigma; #U5378) in 20 mM 4-Hydroxyerhylpiperazine-1-propanesulfonic acid (EPPS) (Sigma, #E9502). Lys-C (Wako; #125–05061) was added at a 1:100 w/w ratio to proteins and incubated at 30 °C for 6 h, followed by diluting the 8 M urea into 4 M urea with EPPS buffer. After incubation, the 8 M urea was diluted by EPPS to 1 M and trypsin was added at 1:50 w/w ratio and incubated at 37 °C overnight. Afterwards, the samples were acidified by TFA (Sigma; #302,031-M), cleaned using Sep-Pak (Waters; Cat# WAT054960) and dried. Samples were loaded with buffer A (0.1% FA in water) onto a 50 cm EASY-Spray column connected to the EASY-nLC 1000 (Thermo; #LC120) and eluted with a buffer B gradient. Mass spectrometry were acquired with an Orbitrap Q Exactive HFX Orbitrap instrument (Thermo). The raw data collected from LC–MS were analyzed by MaxQuant, version 1.5.6.5. STRING version 10.5 tool (http://string-db.org) was used for proteins network analysis. Data were processed by Excel, R and Prism. Each sample was performed triple replicates.

### Flow cytometric analyses

Glioblastoma cells were stained with 25 nM MitoTracker® Deep Red FM for 20 min in the dark at 37 °C for mitochondrial mass measurements. To monitor the production of reactive oxygen species (ROS), the cells were stained with 2′,7′-Dichlorofluorescin diacetate (DCFH-DA) (20 µg/mL, 1:5000 in use; D6883, Sigma) at 37 °C in the dark for 20 min. After a thorough wash with DPBS, cancer cells were analysed using an FC500 flow cytometer (Beckman Coulter; Hialeah, FL, USA) Data analyses were performed using the FlowJo software.

### Western blot

U87 and U38 cells were lysed in EBC buffer (50 mM Tris, pH8.0, 120 mM NaCl and 0.5% NP-40) containing protease inhibitors and phosphatase inhibitors. After electrophoretic separation and transfer of proteins, PVDF (or nitrocellulose) membranes were incubated with the following primary antibodies overnight at 4 °C: rabbit anti-human mitochondrially Encoded Cytochrome C Oxidase II (MTCO2) (Cat#ab91317, Abcam), rabbit anti-human mitochondrial transcription factor A (TFAM) (Cat#8076, CST), rabbit anti-human phospho-VEGFR2(Cat#AP0382, abclonal), rabbit anti human VEGFR2 (Cat#26415–1-AP, proteintech), rabbit anti-human phospho-Akt (Thr308) (Cat#4056, CST) and rabbit anti-human Akt Serine-Threonine Kinase (Cat#9272, CST), rabbit anti-human Phospho-Peroxisome Proliferator-Activated Receptor Gamma Coactivator 1-Alpha (Phospho-PGC1α (S571)) (Cat#AF6650, R&D Systems), mouse anti-human Anti-PGC1α (Cat#ST1202, Millipore), mouse anti-human GAPDH (Cat#ab8245, Abcam). After through washing, the membranes were probed with Invitrogen anti-rabbit IgG (H + L) highly cross-adsorbed secondary antibody (Cat#A16035) and anti-mouse IgG (H + L) highly cross-adsorbed secondary antibody (Cat#A16017). Immunoreactive proteins were visualized by an enhanced chemiluminescence kit (ECL Plus, GE Healthcare). The blotting images were analyzed using ImageJ (NIH).

### Confocal microscopy

For confocal microscopic imaging, cancer cells were cultured on glass coverslips. Mitochondrial staining was carried out using 200 nM MitoTracker Red CMXRos (M7512, ThermoFisher) at 37 °C for 30 min. Afterwards, the cells were fixed for 10 min in prewarmed 4% paraformaldehyde, and then washed with pre-warmed phosphate-buffered saline (PBS). Mitochondrial ROS production was visualized by MitoSox RED (M36008, ThermoFisher; 37 °C, 30 min), while PGC1α staining using PGC1α antibody (Millipore, ST1202). The coverslips were mounted using the ProLong Diamond Antifade Mountant containing 4′6-diamidino-2-phenylindole (DAPI) (Thermo Fisher, Cat#: P36962) for nuclear staining (22 °C, 30 min). Images were acquired with a Leica TCS SP2 AOBS (Acoustico Optical Beam Splitter) inverted laser scanning confocal microscope equipped with a 63 × water immersion objective (HCX PL APO 63.0 × 1.20 water corrected). DAPI and MitoTracker Red CMXRos were excited with Ultraviolet or 359 nm and 579 nm lasers, respectively. Images processing were carried out with ImageJ software (imagej.nih.gov).

### Cell cycle analyses

U87 and U38 cells were seeded into 12-well plates at 5 × 10^4^ cells per well, followed by adding Ki8751 (0, 2.5 and 5 µM) after 24 h-culure with triplicates on each condition. The cells were harvested by using Trypsin–EDTA solution, washed by PBS, then fixed in 1 mL ice-cold 70% ethanol and kept at − 20 °C for more than 2 h. Before analysis, cells were stained with 300 µL PI/RNase staining buffer (BD Pharmingen; Cat# 550,825) for 15 min at room temperature. Cell cycle analyses were performed using NovoCyte flow cytometer (ACEA Bioscience; San Diego, CA, USA).

### Cellular senescence assay

U87 and U38 cells were cultured with 6-well plates (2 × 10^5^ cells per well) for 24 h. Ki8751 (0, 2.5 and 5 µM) was then added and further cultured for 48 h. Thereafter, the cells were stained using a senescence cell staining kit (CS0030, Sigma). Briefly, cells were fixed and washed. The cells were then stained with X-gal staining mixture at 37 °C for 24 h. After this time, the cells were observed and photographed under a fluorescent microscope. The percentages of senescent cells were calculated by blue-stained cell counts divided by the total cell number.

### shRNAs and lentivirus infection

Lentiviral mission shRNA clones against VEGFR2 (TRCN0000001686, TRCN0000001687 and TRCN0000001688) were purchased from Sigma, named sh#1, #2, #3, respectively. U87 and U38 cells were infected with lentivirus for 24 h, and then selected for one week with puromycin (2 µg/mL). Afterwards, the cancer cells were harvested for Western blot of VEGFR2 expression and flow cytometric analysis of mitochondrial mass.

### Oxygen consumption measurements

Metabolic analyses of U87 cells were preformed using the Seahorse XFp analyser (Agilent; North Billerica, MA, USA). Cells (22,000/well) were seeded in an XFp 96-well plate, and then treated with vehicle or Ki8751(2.5 µM). After incubation for 24 h, cells were used for Mito Stress assay. Cells were first washed and preincubated for one hour with Seahorse XF DMEM medium (pH7.4) (Agilent) supplemented with 1 mM sodium pyruvate, 10 mM glucose, and 2 mM L-glutamine (Sigma). Oxygen consumption rate (OCR) was analyzed at basal conditions and after sequential injections of oligomycin (1 µM), carbonyl cyanide-4-(trifluoromethoxy) phenylhydrazone (FCCP; 1 µM), and antimycin/rotenone (0.5 µM). All metabolic assays were normalized to the total protein content.

### Cytoplasmic and nuclear extractions

U87 cells were harvested after treatment by DMSO or Ki8751, or after transfection by shRNA (shSCR, sh#1, #2, #3). Cell pellets were washed once with PBS, and preparation of cytoplasmic extract and nuclear extract were then conducted by using NE-PER™ Nuclear and Cytoplasmic Extraction Reagents (#78833, Thermo Fisher Scientific) according to the manufacturer’s instructions. The protein levels were quantified and the same amounts of protein (50 μg cytoplasmic proteins and 15 μg nuclear proteins) were loaded on SDS-PAGE gels and run Western blot to compare PGC1α expression (clone 4C1.3; ST1202; Millipore, Hayward, CA, USA), where GAPDH (ab22555; 1:1,000; Abcam, Cambridge, UK) and LaminB1 (ab16048; 1:1,000; Abcam) were used to assess cytoplasmic and nuclear protein input levels, respectively.

### Statistics

Data are presented as mean ± SEM. Comparisons between treatments were analysed by one-way ANOVA followed by Tukey’s multiple comparison test where appropriate using GraphPad 6 (GraphPad Software, San Diego, CA, USA). *p* < 0.05 was deemed statistically significant.

## Results

### VEGFR2 blockade impedes glioblastoma cell proliferation and induces cell apoptosis

**Fig. 1:**
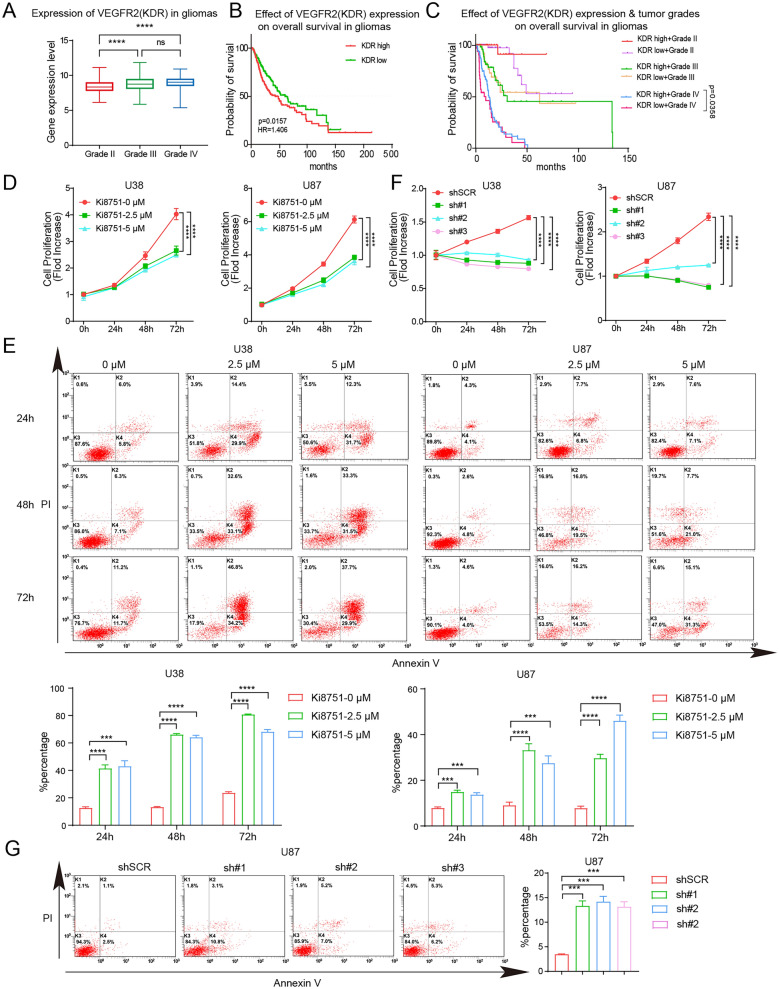
VEGFR2 inhibition decreases glioblastoma cell proliferation and increases cell apoptosis. **A** *VEGFR2* transcriptomic levels of cancer cells were higher in grade 3 and grade 4 gliomas than grade 2 gliomas based on TCGA database. **B** Higher *VEGFR2* expression levels of glioblastoma cells are linked to a worse survival in gliomas patients. **C** *VEGFR2* expression levels of glioblastoma cells are not directed linked to the survival rate in gliomas patients of different grades. **D** Cell viability in U38 and U87 cells as assessed by using a CCK-8 assay in the presence of vehicle (0.01% DMSO) or Ki8751 treatment (2.5 µM and 5 µM) for 24 h, 48 h and 72 h. The fold changes were normalized by the optical density (OD) values at the start time point 0. Mean ± SEMs are plotted. ****p < 0.0001 as compared to vehicle, n = 3. **E** Cell apoptosis analyses U38 and U87 cells by Annexin V/PI staining in the presence of vehicle (0.01% DMSO) or Ki8751 treatment (2.5 µM and 5 µM) for 24 h, 48 h and 72 h. The bar graphs show the percentages of apoptosis cells. Mean ± SEMs; ***p < 0.001, ****p < 0.0001, as compared to vehicle, n = 3. **F** Cell viability of U38 and U87 cells assessed by the CCK-8 assay. *VEGFR2* knockdown of U38 and U87 cells was performed using specific shRNA #1, #2 and #3, as well as the control shSCR. **G** Cell apoptosis analyses of U87 cells by Annexin V/PI staining. The bar graph shows the percentages of cell apoptosis. Mean ± SEMs; ***p < 0.001 as compared to shSCR, n = 3

Since VEGFR2 expression has been reported to contribute to angiogenesis and cell proliferation in glioblastoma, *VEGFR2* expression in cancer cells among glioblastoma patients was therefore collected from the public database TCGA. According to WHO grading criteria, 223 patients of grade II, 245 patients of grade III, and 168 patients of grade IV were identified. Figure 1A shows that grade III and grade IV patients had higher *VEGFR2* (*KDR*) expression on tumor cells than that in grade II patients. Higher *VEGFR2* expression were linked to poorer survival as compared to the patients with lower *VEGFR2* expression (Fig. 1B), although the correlation of *VEGFR2* expression and prognosis showed no significance in each grade except in grade IV (Fig. 1C).

To further elucidate the effect of VEGFR2 inhibition on glioblastoma cell viability, we treated glioblastoma cell lines with Ki8751, a VEGFR2-specific inhibitor. Firstly, drug titration assay was performed, and 2.5 and 5 μM were chosen as the two concentrations for further investigations, which decreased cell counts by approximately 50% (Additional file 1: Fig. S1A). Hence, Ki8751 treatments (0, 2.5 and 5 μM) were applied to the cultured glioblastoma cells U87 and U38 respectively. Cell proliferation was monitored at 24, 48 and 72 h post treatment by CCK8 assay. As shown in Fig. 1D, U38 cells cultured with vehicle (control) proliferated over time, especially after 72 h with nearly fourfold increase of cell numbers, whereas cell proliferation was clearly and similarly hampered in the presence of 2.5 µM and 5 µM Ki8751 (Fig. 1D, left panel). Similar results were seen in U87 cells (Fig. 1D, right panel). In addition, cell apoptosis of U38 and U87 cells, as indicated by Annexin V-FITC and PI staining, were markedly and time-dependently increased by Ki8751 treatments (Fig. 1D).

To clarify the off-target possibility of Ki8751, *VEGFR2* expression of U38 and U87 cells were knocked down using shRNAs (sh#1, #2, #3). Figure 1F shows that all three shRNAs decreased cell proliferation in both U38 and U87 cells. shRNA knockdown also induced the apoptosis of U87 and U38 cells (Fig. 1G and Additional file 1: Fig. S1B). Taken together, these results indicate that VEGFR2 expression was increased in high-grade glioblastoma patients, and that VEGFR2 inhibition exerts anti-proliferation and pro-apoptosis activities in glioblastoma cells.

### Ki8751 treatment increases the expression of mitochondrial proteins

**Fig. 2:**
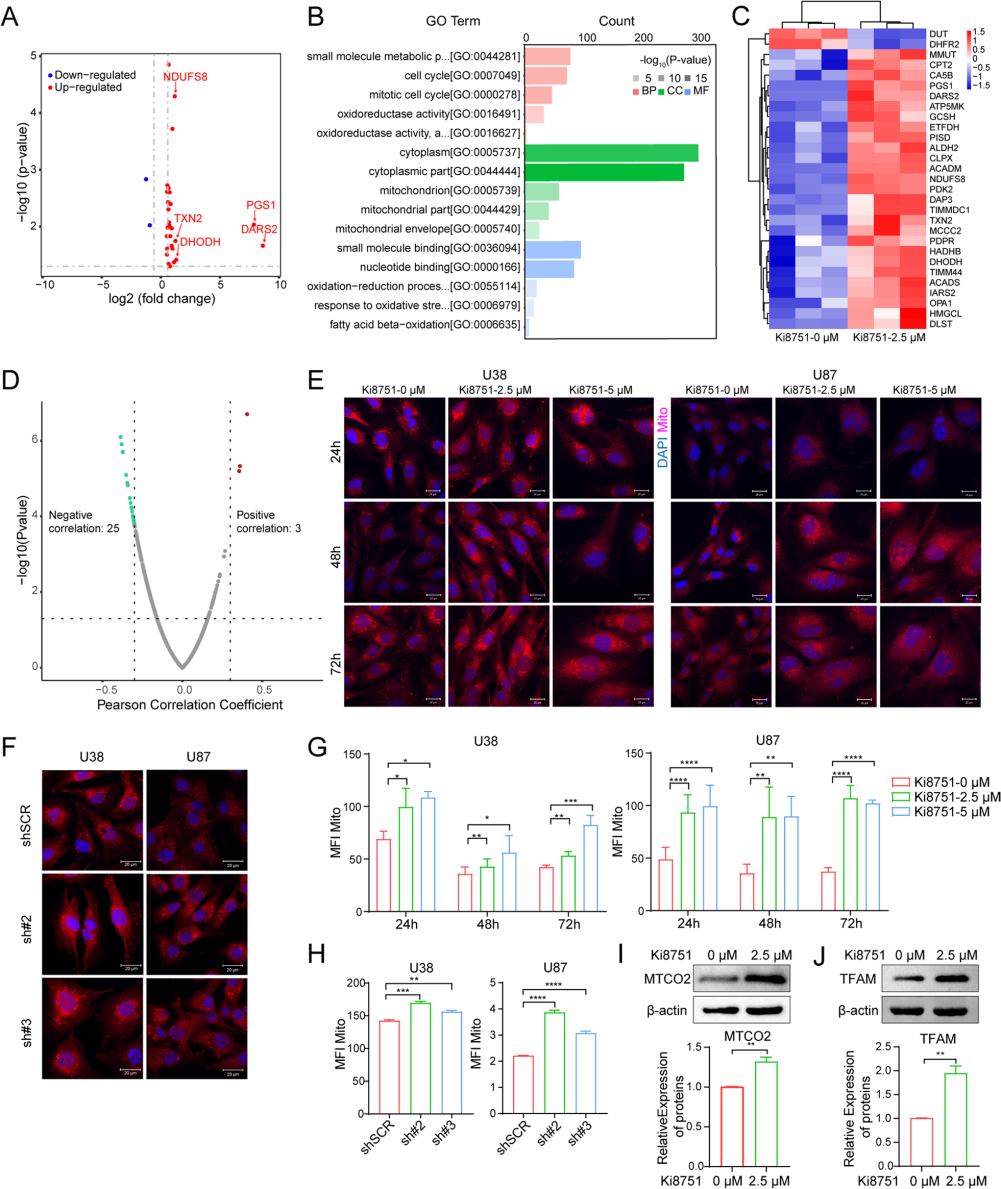
VEGFR2 inhibition up-regulates mitochondria-associated proteins and mitochondrial mass. **A** Volcano plots depicts mitochondrial proteins up- and down-regulated by mass spectrometry in U87 cells treated by 2.5 µM Ki8751 for 24 h. **B** The GO terms of the biological process (BP), cell component (CC) and molecular function (MF) categories enriched of the differentially expressed genes in U87 cells treated by 2.5 µM Ki8751 for 24 h. **C** Heatmap shows the expression level of 27 up-regulated proteins and 2 down-regulated proteins in U87 after Ki8751 treatment. **D** Pearson correlation assay of VEGFR2 expression with mitochondria-related proteins, 25 of them showed negatively correlation with the coefficient less than − 0.3. **E** Fluorescent images displaying the mitochondrial mass of U38 and U87 cells before and after treatment with Ki8751. The cancer cells were stained with MitoTracker Red CMXRos for mitochondria and with DAPI for nuclei. Fluorescent images were acquired using a confocal microscope. Representative images are from three experiments. **F** Fluorescent images displaying the mitochondrial mass in U38 and U87 cells after VEGFR2 knockdown of by shRNAs. Representative images are from three experiments. **G** Mitochondrial mass as assessed by flow cytometry. U38 and U87 cells were treated with 2.5 and 5 µM Ki8751 for 24 h, 48 h, or 72 h. MFI of the mitochondrial dye MitoTracker Red CMXRos are plotted; *p< 0.05, **p< 0.01, ****p< 0.0001, n = 3. **H** Mitochondrial fluorescence intensities in U38 and U87 cells without and with VEGFR2 knockdown by shRNAs. Mean ± SEM, **p < 0.01, ***p < 0.001, ****p < 0.0001, n = 3. **I** Western blot of MTCO2 in U38 and U87 cells without or with Ki8751 treatment. GAPDH was used as the internal control. Representative images are from three independent experiments. **J** Western blot of TFAM in U38 and U87 cells without or with Ki8751 treatment. GAPDH was used as the internal control. Representative images are from three independent experiments

In order to investigate the molecular mechanism by which VEGFR2 inhibition impedes glioblastoma cell proliferation, mass spectrometry was performed in U87 cells after 24 h-treatment with 2.5 µM Ki8751. Detected proteins with the fold of change (FC) equal or greater than 1 with *p* value less than 0.05 in Ki8751-treated cells, as compared to untreated cells, were selected as candidate proteins for further investigated. It was found that 27 mitochondria-related proteins were up-regulated, while 2 mitochondria-related proteins were down-regulated following Ki8751 treatment (Fig. 2A and C). Gene Ontology (GO) analysis showed that these proteins were linked to metabolic process, cell cycle, oxidoreductase activity, mitochondria, response to oxidative process (Fig. 2B), indicating that VEGFR2 inhibition dysregulated the mitochondrial metabolism. To further confirm the correlation of VEGFR2 and mitochondria, the Pearson correlation test was performed based on TCGA dataset, as expectedly, 25 mitochondrial genes were negatively correlated with VEGFR2 gene expression (*r *< − 0.3, *p *< 0.05) (Fig. 2D).

### VEGFR2 inhibition increases mitochondrial mass in glioblastoma cells

To investigate the effect of VEGFR2 blockade on mitochondria biogenesis, mitochondrial mass of U38 and U87 cells was detected with MitoTracker Red CMXRos. The results showed that cell size became larger, and that mitochondrial staining was much brighter in the presence of Ki8751, as early as at the time point of 24 h post Ki8751 treatment. Polylobular nuclear cells were also seen, especially in U87 cells (Fig. 2E). Similar increases in cell size and mitochondrial staining brightness were seen after shVEGFR2 intervention (Fig. 2F). When the mean fluorescence intensities (MFI) were quantified, it was clear that VEGFR2 intervention either with Ki8751 treatment or shVEGFR2s elevated mitochondrial MFIs in U38 and U87 cells on all three time points (Fig. 2G and H). The protein level of MTCO2, a component of cytochrome c oxidase and a part of respiratory chain complex IV, and TFAM, mitochondrial transcription factor A, were elevated in U87 cells after Ki8751 treatment. (Fig. 2I and J). TFAM was also found increased in U38 cells after Ki8751 treatment (Additional file 1: Fig. S3B). These data indicated that VEGFR2 inhibition enhanced mitochondria biogenesis.

**Fig. 3:**
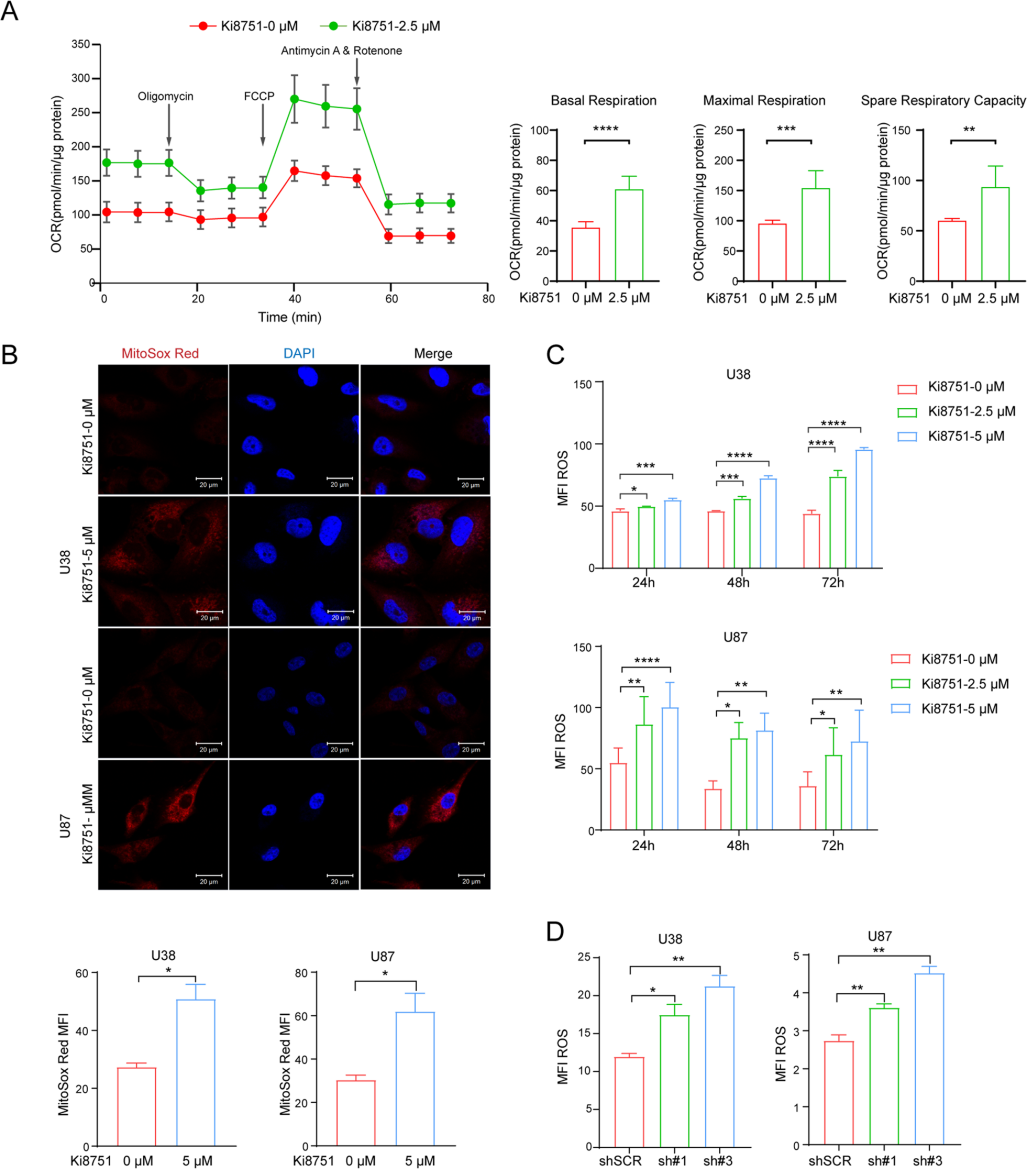
VEGFR2 inhibition by Ki8751 or shRNA increases mitochondrial oxygen consumption and enhances ROS production in glioblastoma cells. **A** OCR in U87 cells without or with 2.5 µM Ki8751 treatment for 24 h measured by Seahorse assay. The bar graphs show the basal OCR, spare respiratory capacity, protein leak and ATP production. Mean ± SEM, n = 3. **B** Fluorescent images displaying the ROS staining in U38 and U87 cells after Ki8751 treatment for 24 h. The bar graph below shows the corresponding ROS mean fluorescence intensity (MFI) of U38 and U87 cells. **C** The bar graph shows ROS MFI of U38 and U87 cells in the presence of vehicle (0.01% DMSO) or Ki8751 treatment (2.5 µM and 5 µM) for 24 h, 48 h and 72 h. Mean ± SEM, *P < 0.05, **P < 0.01, n = 3. **D** The bar graph showing ROS MFI in U38 and U87 after knockdown of VEGFR2 by shRNA

### Ki8751 treatment leads to enhanced cellular oxygen consumption and ROS production

As shown above, VEGFR2 inhibition enhanced mitochondrial biogenesis in glioblastoma cells. We thus assessed the OCRs of U87 cells by Seahorse assay. The basal respiration level and ATP production were increased in U87 cells after Ki8751 treatment for 24 h (Fig. 3A). Redox signaling is essential in mitochondria, and elevated mitochondrial ROS can induce pathological oxidative stress [22]. To delineate the correlation between VEGFR2 inhibition and ROS production, mitochondrial ROS production was monitored by MitoSox Red staining and confocal imaging. As illustrated in Fig. 3B, mitochondrial ROS contents were increased after Ki8751 treatment for 24 h in U38 and U87 cells. Furthermore, intracellular ROS production in U38 and U87 cells were measured by using DCFH-DA fluorescent probes. Flow cytometry analysis indicated that Ki8751 induced an increase in ROS fluorescence levels as early as 24 h post treatment and sustained during 72 h treatment (Fig. 3C). On the other hand, knocking down *VEGFR2* by siRNA or shRNA in both cell lines also resulted in significant increased basal respiration levels and ATP production as well as ROS production (Fig. 3D and Additional file 1: Fig. S2A–D). In short, these data indicate that VEGFR2 inhibition enhances cellular oxygen consumption and promotes ROS production in glioblastoma cells.

### VEGFR2 inhibition arrests cell cycle in a high aneuploid phase and promotes cells into a senescent state

**Fig. 4:**
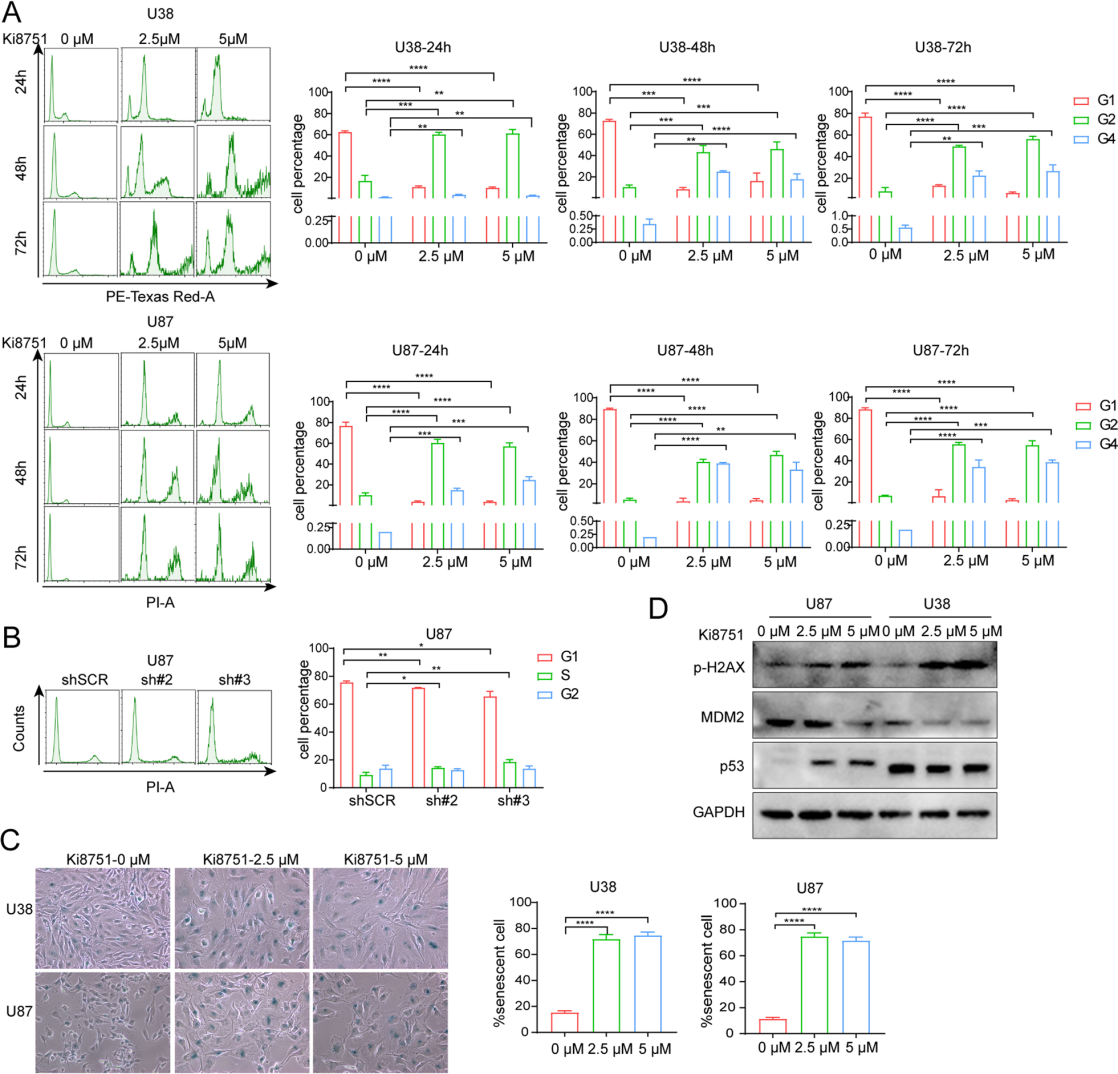
VEGFR2 inhibition by Ki8751 or shRNA induces cell cycle arrest and cell senescence in glioblastoma cells. **A** Cell cycle analyses by PI staining and flow cytometry in U38 and U87 cells after treatment with Ki8751. Bar charts present the percentages of cells in G1, G2, and G4 phase. Mean ± SEM, *p< 0.05, **p < 0.01, ***p < 0.001, n = 3. **B** Cell cycle analyses in U87 cells without or with VEGFR2 knockdown by shRNAs. **C** X-gal staining of senescent cells. U38 and U87 cells were treated with 2.5 and 5 µM Ki8751 for 48h, and then stained using a senescence cell staining kit (CS0030, Sigma). The bar graphs display the mean percentages of senescent cells in the total cells. Mean ± SEM, **** *p* < 0.0001, n = 3. **D** Western blot images demonstrates the protein levels of p-H2AX, MDM2, P53 of U87 and U38 cells after the treatment of Ki8751 for 48h

As illustrated in Fig. 2E, larger and polylobular nuclei glioblastoma cells appeared after Ki8751 treatment, indicating an effect on cell division by VEGFR2 inhibition. Cell cycle analyses revealed that Ki8751 treatment robustly increased the percentages of U38 and U87 cells docked in G2/M phase within 24 h, while untreated cells were predominantly retained in G1 phase. Notably, increasing numbers of cells appeared in a higher aneuploid phase than G2 phase, herein named G4 phase, after prolonged Ki8751 treatment for 48 h and 72 h in both U38 and U87 cells (Fig. 4A). Furthermore, we tested VEGFR2 knockdown by using shRNA in U87 and found a slight decrease of cell proportion in G1 phase and increase in S phase (Fig. 4B). These findings demonstrated that VEGFR2 inhibition caused cell cycle arrested in high aneuploid phases (G2/M and G4) in glioblastoma cells.

Since cell senescence and mitochondrial dysfunction are two important processes in response to various stressors, we then tested the cell senescence state after VEGFR2 inhibition. β-gal staining positive senescent cells were elevated by more than four folds after addition of Ki8751 (2.5 and 5 μM) for 48 h (Fig. 4C). In consistence, western blot results showed the increased expression of p-H2AX, a marker of cell senescence. Moreover, the expression of P53 was increased, but MDM2 expression decreased by Ki8751 treatments, indicating that P53 pathway was involved in this senescent process (Fig. 4D). These data demonstrated that VEGFR2 inhibition induced glioblastoma cells into a senescent state.

### VEGFR2 inhibition exerts its effects via Akt/ PGC1α/TFAM signaling pathway

**Fig. 5:**
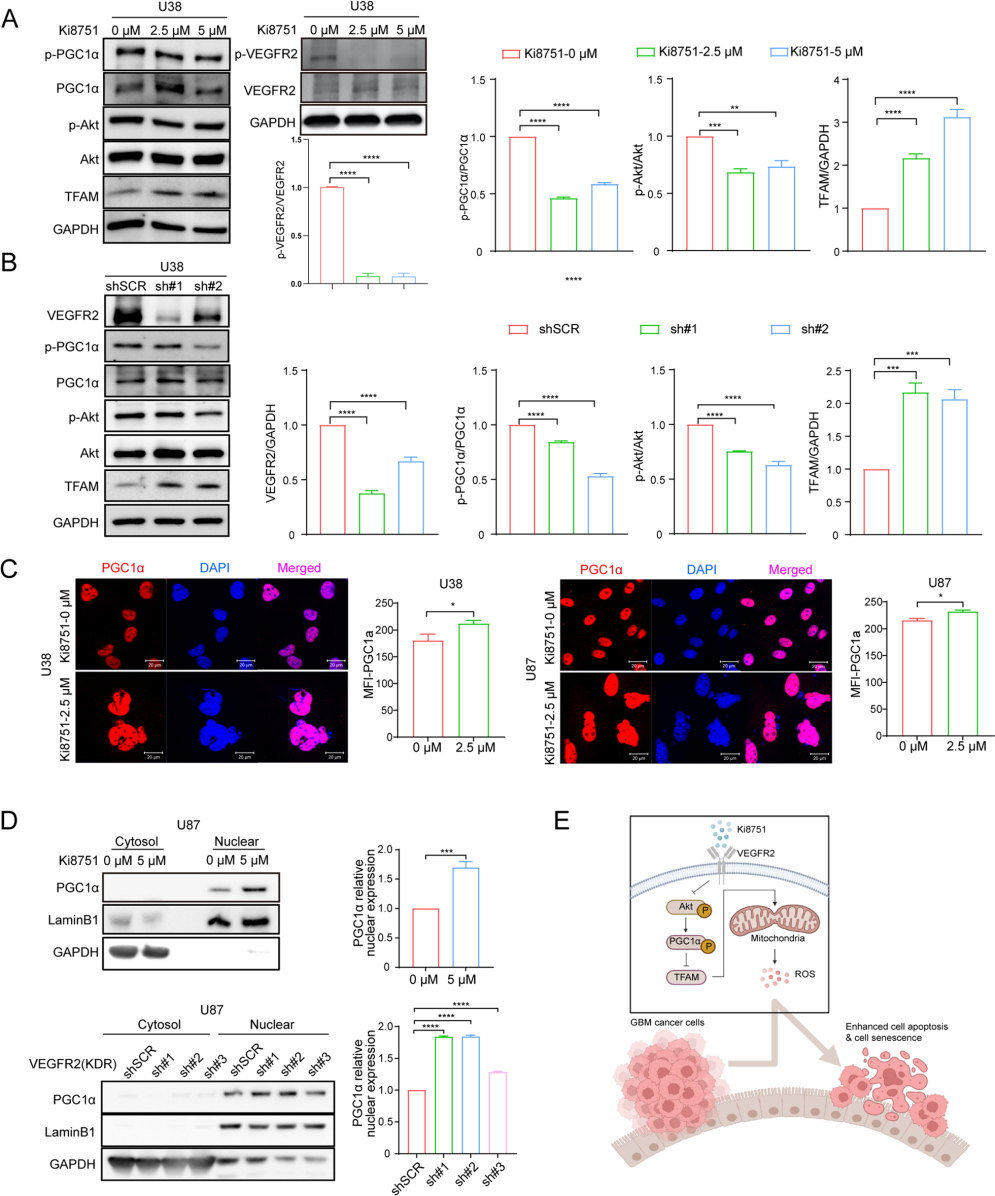
VEGFR2 inhibition by Ki8751 interferes VEGF intracellular signaling in glioblastoma cells. **A** Western blot images showing the protein levels of p-VEGFR2, VEGFR2, p-PGC1α, PGC1α, p-AKT, AKT, TFAM of U38 cell in the absence or presence of Ki8751treatment for 48 h. The bar graphs plot the relative intensities of p-Akt and p-PGC1α. Mean ± SEM, ***p < 0.001, ****p < 0.0001 as compared to vehicle treatment. **B** Western blot images of p-PGC1α, PGC1α, p-AKT, AKT, and TFAM immunoreactive bands of U38 cells with VEGFR2 knockdown by shRNAs. Mean ± SEM, ****p < 0.0001 as compared to the control shSCR. **C** Immunofluorescence images demonstrate the cellular location of PGC1α staining in U38 and U87 cells without or with Ki8751 treatment for 48 h. DAPI was used as a nuclear location marker. Bar charts depict PGC1α fluorescence intensities without or with Ki8751 treatment. Mean ± SEM, *p < 0.05 as compared to vehicle, n = 3. **D** Western blot images showing the protein level of PGC1α in the cytosol and nucleus of U87 cells after treatment with Ki8751 or shRNA knockdown. LaminB1 and GAPDH were used as input control for nuclear protein or cytoplasmic protein, respectively. Bar graphs show the relative expression of nuclear PGC1α in U87 cells. Mean ± SEM, ****p < 0.0001 as compared to the control. **E** Schematic illustration of VEGFR2 inhibition-induced mitochondrial biogenesis signaling and apoptosis in glioblastoma cells

To illustrate how VEGFR2 inhibition affects mitochondria biogenesis, we compared the total and phosphorylated VEGFR2, PGC1α and AKT phosphorylation expression during cell apoptosis induced by Ki8751, which are important intracellular signaling nodes in mitochondria biogenesis. We observed decreased phosphorylated VEGFR2 (p-VEGFR2) levels in both U38 and U87 cells treated by Ki8751 (Fig. 5A and Additional file 1: Fig. S3A). Western blot analyses showed that Ki8751 treatment (2.5 µM, 48 h) reduced phosphorylated AKT (p-AKT, Thr308) of U38 cells, subsequently decreased phosphorylated PGC1α (p-PGC1α) levels (Fig. 5A). TFAM expression was elevated in U38 cells and U87 after Ki8751 treatment (Fig. 5A and Additional file 1: Fig. S3B. Similar results were also seen in U38 cells with shVEGFR2 knockdown (Fig. 5B). Since phosphorylation level of PGC1α affects its nuclear re-localization, PGC1α mobilization was thus monitored with immunofluorescence imaging. Figure 5C shows that PGC1a exhibited stronger staining in the nuclei of both U38 and U87 cells after Ki8751 treatment for 48 h, as compared to control group. Analyses of extracted cytosolic and nuclear PGC1α proteins confirmed that both Ki8751 inhibition and VEGFR2 shRNA knockdown resulted in elevated PGC1α nucleic fraction in U87 cells (Fig. 5D). Furthermore, analyses using TCGA database showed that PGC1α and TFAM expression in Grade 4 gliomas patients were lower than those in Grade 3 patients, and that higher PGC1α and TFAM expression were correlated with better prognosis (Additional file 1: Fig. S3C and D). In short, these data indicated that mitochondria metabolism plays an important role during cell apoptosis and cell senescence induced by VEGFR2 inhibition (Fig. 5E).

## Discussion

The present work demonstrates that VEGFR2 inhibition, either by Ki8751 treatment or siRNA/shRNA knockdown, reduced glioblastoma cell proliferation and promoted cell apoptosis. The effects were exerted by regulating mitochondrial metabolism. Hence, VEGFR2 inhibition decreased AKT and PGC1α phosphorylation, induced PGC1α nuclear translocation, increased mitochondrial markers MTCO2 and TFAM expression, resulting in the elevations of mitochondrial biogenesis, ROS production and OXPHOS respiration.

Mitochondrial metabolism plays a key role in oncogenesis, not only as the major source of ATP, but also the production of ROS and other mediators to activate oncogenic signaling pathways [23]. Normal cells conducted mitochondrial OXPHOS primarily to produce energy. Cancer cells, however, reprogram their metabolism and adapt aerobic glycolysis (Warburg effect) rather than OXPHOS to get more energy [24] and to maintain cell growth and survival. The present study demonstrated that VEGFR2 intervention by Ki8751 or shRNAs induced metabolism reprogramming in glioblastoma cells. This was evidenced by proteomic data that mitochondrial biogenesis/function-related proteins, e.g., PGS1, DARS2, TXN2, DHODH, NDUFS8 et al., were upregulated in U87 glioblastoma cells upon VEGFR2 inhibition by Ki8751 (Fig. 2). In addition, VEGFR2 inhibition promoted mitochondrial biogenesis, seen as increased mitochondrial mass by confocal fluorescence imaging and by flow cytometric mitochondrial fluorescence quantification, as well as increased expression of mitochondrial transcriptional factor, TFAM (Fig. 2). Besides, VEGFR2 blockade elevated the level of MTCO2, a component of cytochrome c oxidase and an enzyme in the mitochondrial electron transport chain that drives oxidative phosphorylation. It should also be noted that VEGFR2 inhibition increased cancer cell size and polylobular nuclear cells, indicating the endomitosis of glioma cells, which is supported by another piece of evidence demonstrating that VEGFR inhibitor induced misalignment of chromosomes and caused delay in M-Phage progression [18]. Furthermore, VEGFR2 inhibition induced glioma cell senescence (Fig. 4), which is consistent with another finding in colorectal cancer cells that mice with low VEGFR2 expression had a higher proportion of senescence cells [13]. In summary, VEGFR2 inhibition elevated mitochondrial mass, increased cellular oxygen consumption, resumed to OXPHOS respiration, and produced more ROS, resulting in cell damage, cell cycle arrest, cell senescence, and activating cell apoptosis processes. These findings highlight the notion that inhibition of VEGF receptors can not only reduce angiogenesis in tumour but also reprogramme cancer cells into OXPHOS respiration and subsequently enhance apoptosis of cancer cells.

Better understanding of the mechanisms underlying VEGFR2 inhibition-induced mitochondrial biogenesis is of great importance for therapeutic potentials of VEGF receptor intervention. There are three core signaling pathways on the downstream of VEGFR2, including PI3K (phosphatidylinositol 3-kinase)/Akt pathway, Raf/MEK (mitogen-activated protein kinase)/MAPK (mitogen-activated protein kinase) pathway, and Src/FAK (focal adhesion kinase) pathway [25]. AKT phosphorylation was found to be enhanced and essential for endothelial proliferation upon VEGFA-VEGFR2 ligation [26]. Phosphoinositide 3-kinase (PI3K)-Akt signaling pathway is critical for tumorigenesis and is one of core signaling pathways in the downstream of VEGFR2. VEGFR2 ligation leads to activation of PI3K [13]. The latter phosphorylates phosphatidylinositol-4,5-bisphosphate (PIP2) and converts PIP2 to PIP3. Afterwards, PIP3 translocates to the membrane and activates phosphatidylinositol dependent kinases (PDK). PDK1 phosphorylates AKT on Thr308 what is both necessary and sufficient for AKT activation [27]. AKT subsequently phosphorylates several targets, including glycogen synthase kinase 3 β (GSK3β), another multifunctional serine/threonine kinase. GSK3β binds on PGC1α and phosphorylates PGC1α to induce intranuclear proteasomal degradation [28, 29]. In addition, high levels of phosphorylated AKT (p-AKT) and phosphorylated GSK3β (Tyr216) were correlated to a poor prognosis in glioblastoma, and silencing of GSK3β induced cell apoptosis and increased the levels of the tumor suppressors p53 and p21[30, 31]. Hence, VEGFR2 ligation activates PI3K, and the VEGFR2-PI3K-PIP3-AKT-GSK3β-PGC1α axis mediates the metabolic reprogramming process in glioblastoma. PGC1α is a mitochondria biogenesis factor, and it is associated with multiple aspects of tumour cell biology. In melanoma, PGC1α positive cells had a stronger mitochondrial energy metabolism and protected cells from oxidative stress [32]. PGC1α also suppressed tumour metastasis in melanoma and prostate cancer [33, 34]. In the present study, VEGFR2 inhibition in glioblastoma cells was shown to decrease the levels of AKT phosphorylation and thus PGC1α phosphorylation (Fig. 5). It has been reported that PGC1α phosphorylation attenuates PGC1α degradation, subsequently suppresses mitochondrial biogenesis and confers radiation resistance in glioma [35]. Aligning with those findings, VEGFR2 inhibition in our study was found to enhance the translocation of PGC1α into nucleus and promote mitochondrial biogenesis in glioblastoma cells (Figs. 2 and 5). While it remains to be validated if VEGFR2 inhibition would increase the radiation sensitivity in glioma patients, VEGFR2 inhibition has been shown to enhanced cell sensitivity to chemotherapy in a PGC1α-dependent manner in acute myeloid leukaemia [36]. Collectively, these findings indicate that the combination of metabolism intervention and anti-cellular drugs may enhance anticancer treatment efficiency.

In the present study, we found that VEGFR2 inhibition elevated mitochondrial biogenesis and increased OXPHOS respiration, resulting in suppressing cell proliferation and promoting cell apoptosis. There is an earlier report showing that temozolomide treatment increased mitochondria size and OXPHOS levels in glioblastomas and resulted in temozolomide resistance, and that the interruption of mitochondria fusion process downregulated OXPHOS level and sensitized GBM cells to temozolomide [37]. Thus, the impact of elevated OXPHOS levels for glioblastoma treatment may be complex. A number of studies reported that glioblastoma stem cells (GSCs), one important population that resists current therapies, relied on OXPHOS. Manipulating cancer cell metabolism by inhibiting mitochondrial OXPHOS may thus improve radiation and chemotherapy response and can serve as a therapeutic option for glioblastoma. Intervention of mitochondrial translation suppressed GSCs and improved their radiation response [38]. The drugs that inhibit mitochondrial translation caused mitochondrial dysfunction by inducing ferroptosis [39], and the combination of anti-parasitic drugs with radiotherapy potently enhanced radiosensitivity of high-grade glioma [40], indicating the importance of OXPHOS in radioresistant of glioblastoma. In the present study, VEGFR2 inhibition was shown to promote OXPHOS and ROS production and lead to cell apoptosis. The different responses of individual cancer cells to increased OXPHOS might be due to the different metabolic signaling pathways dominated in different cancer cells. Nevertheless, the differences remind us to study further on mitochondrial translation and ferroptosis in GSCs after VEGFR2 inhibition, and to validate the therapeutic effects by the combination of VEGFR2 inhibition and chemo-/radio-therapies.

VEGFR2 signaling may exert context-dependent impacts across diverse cancer types, such as their impact on mitochondrial metabolism. In acute myeloid leukemia, VEGFR2 inhibition enhanced cell sensitivity to chemotherapy in a PGC1α-dependent manner with increased mitochondrial mass [36], while the depletion of PGC1α abolished such induction of mitochondrial metabolism and chemosensitization in response to VEGFR2 inhibition. In ovarian cancer cells, VEGFR2 blockade suppressed glycolysis by inhibiting the VEGFR2-AKT1-GSK3β-SOX5-GLUT4 signaling pathway [41]. VEGFR2-FAK/AKT-STAT3 signaling axis has also been shown to induce chemotherapy of ovarian cancer cells by regulating angiogenesis and glycolysis [42]. In our previous study, we found that VEGFR2 blockade hampered breast cancer cell proliferation via AKT-PGC1α pathway and increased mitochondria biogenesis [19]. Collectively, these studies indicate that VEGFR2/PI3K/AKT signaling may influence multiple aspects of mitochondrial metabolisms in different types of cancer cells.

There are some limitations in the present study. Our work clearly showed that VEGFR2-inhibition exerts anti-proliferative effect by promoting mitochondrial biogenesis. Albeit not performed in the present study, additional observations of the impact on mitochondrial biogenesis and cell proliferation by the treatment with mitochondrial inhibitors, such as metformin, menadione or tigecycline, would be helpful to add further evidence of the involvement of mitochondria. Similarly, we have identified the involvement of Akt-PGC1α-TFAM signaling pathway during VEGFR2 inhibition; the application of AKT or PGC1α activators or inhibitors would be helpful to further confirm the pathway. Moreover, although beyond the capacity of the present study, future studies are warranted to illustrate the anticancer effects by combining VEGFR2 inhibitor and chemotherapy in glioma cells and to validate our findings in vivo using a xenograft experimental setup.

In conclusion, VEGFR2 inhibition decreases cell proliferation, but promotes cell senescence and apoptosis in glioblastoma cells. The anti-cancer effect is exerted via Akt-PGC1α-TFAM-mitochondria biogenesis signaling that reprograms cancer cell metabolisms prone to mitochondrial OXPHOS respiration and ROS production, and subsequently cancer cell apoptosis (Fig. 5E). Our findings suggest that VEGFR2 inhibition and its regulation on mitochondrial metabolism are potential intervention sites for alternative anticancer treatments.

### Supplementary Information

**Additional file 1: Figure S1.** VEGFR2 inhibition increases cell apoptosis. **A** Ki8751 dose response curve as assessed by cell viability of U38 and U87 cells. **B** Cell apoptosis analyses by Annexin V/PI staining in U38 cells. The bar graph depicts U38 cell apoptosis percentages per VEGFR2 knockdown by shRNAs. **Figure S2.** VEGFR2 inhibition by siRNA increases mitochondrial oxygen consumption and enhances ROS production in glioblastoma cells. **A** OCR in U87 cells after knockdown of VEGFR2 by siRNA for 48 h measured by Seahorse assay. The bar graphs show the basal OCR, spare respiratory capacity, protein leak and ATP production. Mean ± SEM, n = 3. **B** Fluorescent images displaying the ROS staining in U38 and U87 cells after knockdown of VEGFR2 by siRNA for 48 h. **C** The bar graph shows the corresponding ROS mean fluorescence intensity (MFI) of U38 and U87 cells. **Figure S3. **VEGFR2 inhibition by Ki8751 interferes expression of pVEGFR2 and TFAM and the higher expression of PPARGC1A and TFAM indicates good survival. **A** Western blot images demonstrate the protein levels of pVEGFR2 and VEGFR2 of U87 cells after the treatment of Ki8751 for 48 h. **B** Western blot images demonstrate the protein levels of TFAM of U38 and U87 cells after the treatment of Ki8751 for 48 h. **C** Transcripts of *PPARGC1A* in grade 2, 3 and 4 gliomas and their impact on the survival curve of gliomas patients. **D** Transcripts of *TFAM* in grade 2, 3 and 4 gliomas and their impact on the survival curve of gliomas patients.

## Data Availability

The datasets used and/or analysed during the current study are available from the corresponding author on reasonable request.

## Abbreviations

ATCC: American Type Culture Collection
DCFH-DA: 2′,7′-Dichlorofluorescin diacetate
DMEM: Dulbecco’s Modified Eagle Medium
DMSO: Dimethyl sulfoxide
DPBS: Dulbecco’s phosphate buffered saline
FBS: Fetal bovine serum
GAPDH: Glyceraldehyde-3-phosphate dehydrogenase
GO: Gene Ontology
KDR: Kinase insert domain receptor
MDM2: Murine double minute 2
MEM: Minimum Essential Medium
MS: Mass spectrometry
MTCO2: Mitochondrially Encoded Cytochrome C Oxidase II
OCRs: Oxygen consumption rates
OXPHOS: Oxidative phosphorylation
PGC1α: Peroxisome proliferator-activated receptor gamma coactivator 1-alpha
PI: Propidium iodide
PPARGC1A: Peroxisome proliferator-activated receptor gamma coactivator 1-alpha
ROS: Reactive oxygen species
TCGA: The Cancer Genome Altas
TFAM: Mitochondrial transcription factor A
VEGFR2: Vascular endothelial growth factor receptor 2
WHO: World Health Organization
