# Supplementary material for: VEGFR2 blockade inhibits glioblastoma cell proliferation by enhancing mitochondrial biogenesis
Source: J Transl Med. 2024 May 3;22:419. doi: 10.1186/s12967-024-05155-1 (PMC11067099; doi:10.1186/s12967-024-05155-1)

**Supplemental information**

VEGFR2 blockade inhibits glioblastoma cell proliferation by enhancing mitochondrial biogenesis

Min Guo^1#^, Junhao Zhang^2,3#^, Jiang Han^4#^, Yingyue Hu^4#^, Hao Ni^2,5^, Juan Yuan^6^, Yang Sun^7^, Meijuan Liu^4^, Lifen Gao^7^, Wangjun Liao^3^, Chunhong Ma^7^, Yaou Liu^1^, Shuijie Li^4^, Nailin Li^2^

^1^Department of Radiology, Beijing Tiantan Hospital, Capital Medical University, Beijing, China

^2^Department of Medicine-Solna, Cardiovascular Medicine Unit, Karolinska Institutet, Stockholm, Sweden

^3^Department of Oncology, Nanfang Hospital, Southern Medical University, Guangzhou, China

^4^Department of Biopharmaceutical Sciences and National Key Laboratory of Frigid Zone Cardiovascular Diseases （NKLFZCD）, College of Pharmacy, Harbin Medical University, Harbin, China.

^5^Department of Gynaecology and Obstetrics, Nanfang Hospital, Southern Medical University, Guangzhou 510515, China

^6^Department of Cell and Molecular Biology, Karolinska Institutet, Stockholm, Sweden

^7^Department of Immunology and Shandong University-Karolinska Institutet Collaborative Laboratory, Shandong University Cheeloo Medical College, School of Basic Medicine, Jinan, China

Running title: VEGFR blockade inhibits glioblastoma cell growth

# These authors contributed equally to this work

**Corresponding Authors:**

Min Guo, MD, PhD, Department of Radiology, Beijing Tiantan Hospital, Capital Medical University, Beijing, China. email: guomin04@126.com; ORCID ID: 0000-0002-2291-1230

Shuijie Li, PhD, Department of Biopharmaceutical Sciences and National Key Laboratory of Frigid Zone Cardiovascular Diseases（NKLFZCD）, College of Pharmacy, Harbin Medical University, Harbin, China; email: shuijie.li@hrbmu.edu.cn ; ORCID ID: 0000-0003-4538-8996

Nailin Li, MD, PhD, Department of Medicine-Solna, Division of Cardiovascular Medicine, Karolinska University Hospital-Solna, 171 76 Stockholm, Sweden; email: [nailin.li@ki.se](mailto:nailin.li@ki.se); ORCID ID: 0000-0002-3848-7251

# Supplemental Figure Legends

**Figure S1. VEGFR2 inhibition increases cell apoptosis.** (A) Ki8751 dose response curve as assessed by cell viability of U38 and U87 cells. (B) Cell apoptosis analyses by Annexin V/PI staining in U38 cells. The bar graph depicts U38 cell apoptosis percentages per VEGFR2 knockdown by shRNAs.

**Figure S2.** **VEGFR2 inhibition by siRNA increases mitochondrial oxygen consumption and enhances ROS production in glioblastoma cells** (A) OCR in U87 cells after knockdown of VEGFR2 by siRNA for 48h measured by Seahorse assay. The bar graphs show the basal OCR, spare respiratory capacity, protein leak and ATP production. Mean±SEM, n=3. (B) Fluorescent images displaying the ROS staining in U38 and U87 cells after knockdown of VEGFR2 by siRNA for 48h. (C) The bar graph shows the corresponding ROS mean fluorescence intensity (MFI) of U38 and U87 cells.

**Figure S3. VEGFR2 inhibition by Ki8751 interferes expression of pVEGFR2 and TFAM and the higher expression of PPARGC1A and TFAM indicates good survival.** (A) Western blot images demonstrate the protein levels of pVEGFR2 and VEGFR2 of U87 cells after the treatment of Ki8751 for 48h.(B) Western blot images demonstrate the protein levels of TFAM of U38 and U87 cells after the treatment of Ki8751 for 48h. (C) Transcripts of *PPARGC1A* in grade 2, 3 and 4 gliomas and their impact on the survival curve of gliomas patients. (D)Transcripts of *TFAM* in grade 2, 3 and 4 gliomas and their impact on the survival curve of gliomas patients.


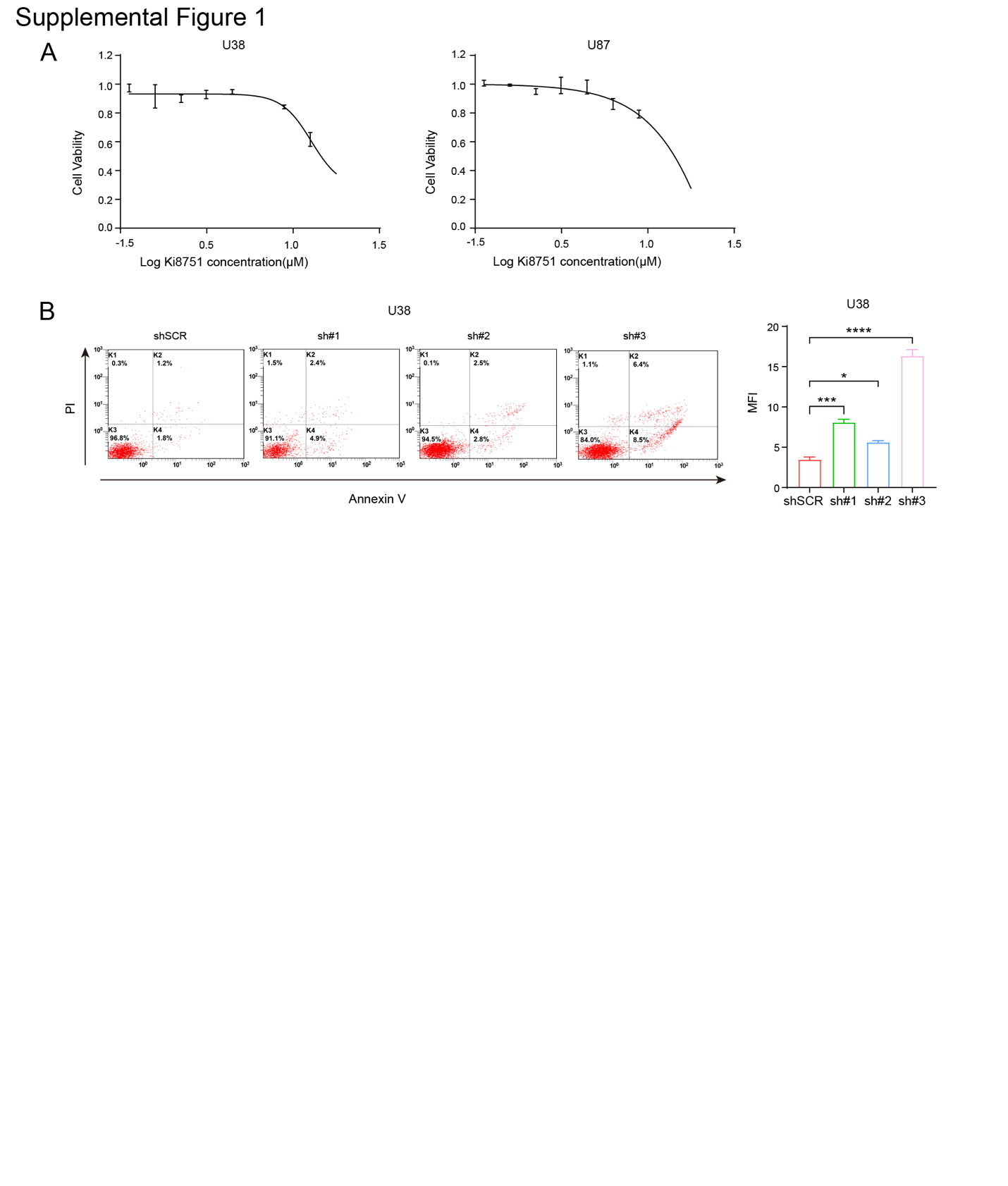


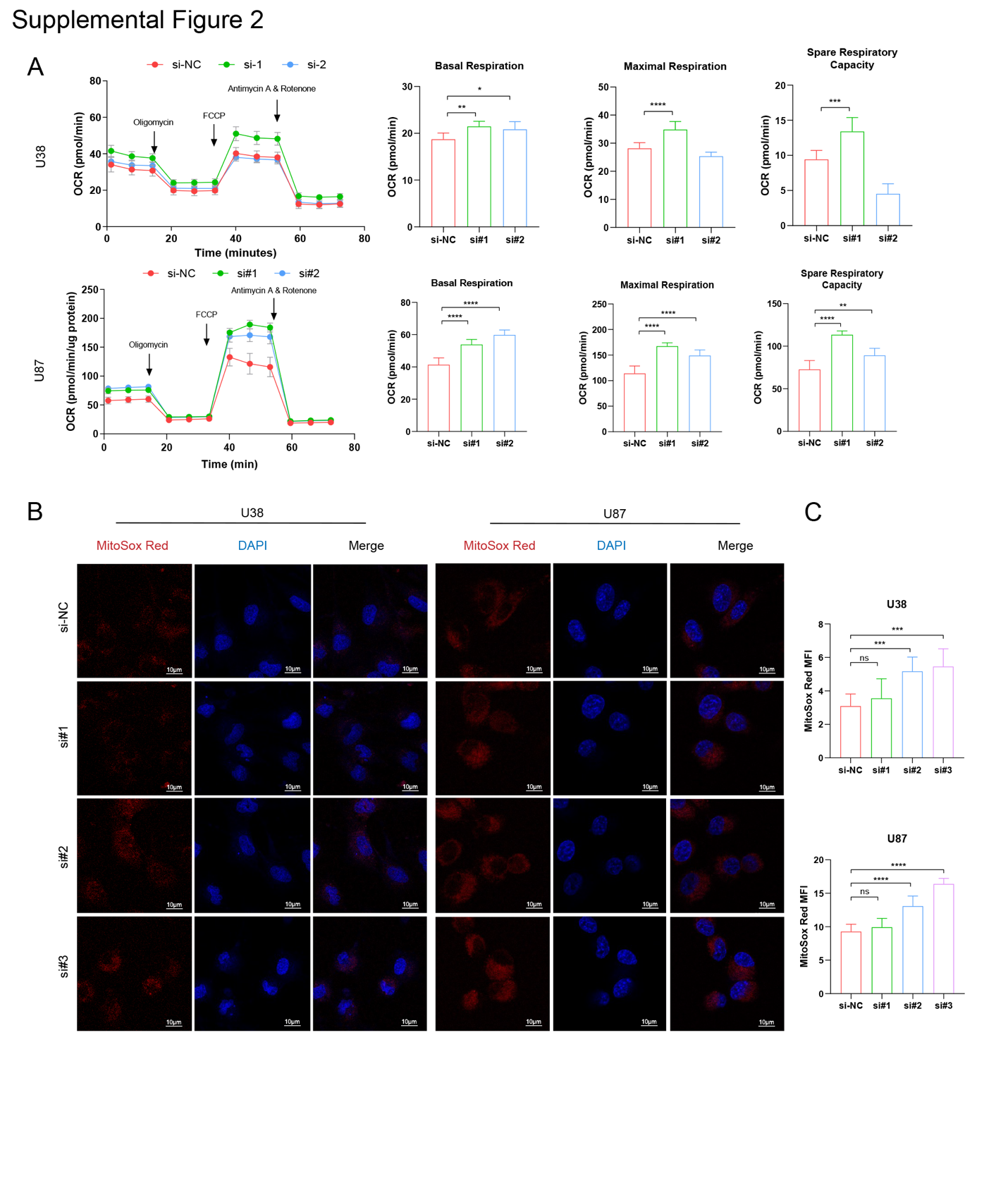


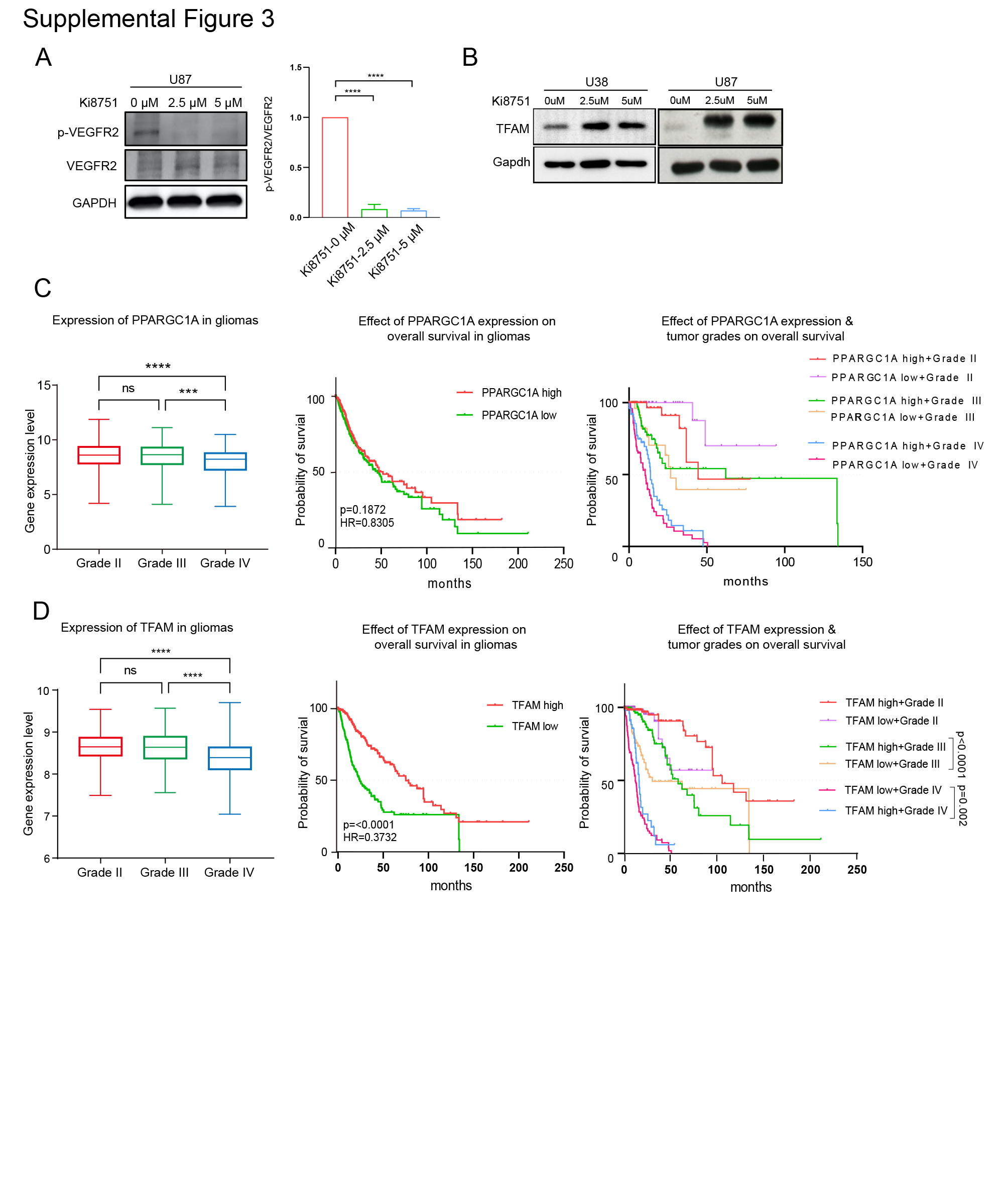

Supplement: Supplementary file 1 — Additional file 1: Figure S1. VEGFR2 inhibition increases cell apoptosis. A Ki8751 dose response curve as assessed by cell viability of U38 and U87 cells. B Cell apoptosis analyses by Annexin V/PI staining in U38 cells. The bar graph depicts U38 cell apoptosis percentages per VEGFR2 knockdown by shRNAs. Figure S2. VEGFR2 inhibition by siRNA increases mitochondrial oxygen consumption and enhances ROS production in glioblastoma cells. A OCR in U87 cells after knockdown of VEGFR2 by siRNA for 48 h measured by Seahorse assay. The bar graphs show the basal OCR, spare respiratory capacity, protein leak and ATP production. Mean ± SEM, n = 3. B Fluorescent images displaying the ROS staining in U38 and U87 cells after knockdown of VEGFR2 by siRNA for 48 h. C The bar graph shows the corresponding ROS mean fluorescence intensity (MFI) of U38 and U87 cells. Figure S3. VEGFR2 inhibition by Ki8751 interferes expression of pVEGFR2 and TFAM and the higher expression of PPARGC1A and TFAM indicates good survival. A Western blot images demonstrate the protein levels of pVEGFR2 and VEGFR2 of U87 cells after the treatment of Ki8751 for 48 h. B Western blot images demonstrate the protein levels of TFAM of U38 and U87 cells after the treatment of Ki8751 for 48 h. C Transcripts of PPARGC1A in grade 2, 3 and 4 gliomas and their impact on the survival curve of gliomas patients. D Transcripts of TFAM in grade 2, 3 and 4 gliomas and their impact on the survival curve of gliomas patients. [file 12967_2024_5155_MOESM1_ESM.docx]
